# The impact of movement sonification on haptic perception changes with aging

**DOI:** 10.1038/s41598-021-84581-3

**Published:** 2021-03-04

**Authors:** C. Landelle, J. Danna, B. Nazarian, M. Amberg, F. Giraud, L. Pruvost, R. Kronland-Martinet, S. Ystad, M. Aramaki, Anne Kavounoudias

**Affiliations:** 1 Aix-Marseille Univ, CNRS, Laboratoire de Neurosciences Cognitives, LNC UMR 7291, 3 place Victor Hugo, 13331 Marseille, France; 2 McConnell Brain Imaging Centre, Department of Neurology and Neurosurgery, Montreal Neurological Institute, McGill University, Montreal, QC Canada; 3 Aix-Marseille Univ, CNRS, Institut des Neurosciences de la Timone, INT UMR 7289, Marseille, France; 4 Univ. Lille, Arts et Métiers Institute of Technology, Centrale Lille, Junia,ULR 2697- L2EP, F-59000, Lille, France; 5 Aix-Marseille Univ, CNRS, Perception, Représentations, Image, Son, Musique, PRISM UMR 7061, Marseille, France

**Keywords:** Perception, Cognitive ageing

## Abstract

Combining multisensory sources is crucial to interact with our environment, especially for older people who are facing sensory declines. Here, we examined the influence of textured sounds on haptic exploration of artificial textures in healthy younger and older adults by combining a tactile device (ultrasonic display) with synthetized textured sounds. Participants had to discriminate simulated textures with their right index while they were distracted by three disturbing, more or less textured sounds. These sounds were presented as a real-time auditory feedback based on finger movement sonification and thus gave the sensation that the sounds were produced by the haptic exploration. Finger movement velocity increased across both groups in presence of textured sounds (Rubbing or Squeaking) compared to a non-textured (Neutral) sound. While young adults had the same discrimination threshold, regardless of the sound added, the older adults were more disturbed by the presence of the textured sounds with respect to the Neutral sound. Overall, these findings suggest that irrelevant auditory information was taken into account by all participants, but was appropriately segregated from tactile information by young adults. Older adults failed to segregate auditory information, supporting the hypothesis of general facilitation of multisensory integration with aging.

## Introduction

During haptic exploration, the interaction between our hand and the explored object generates redundant and complementary proprioceptive, tactile, visual, and sometimes, auditory information. This information can be combined and integrated to optimize the final estimation of the object^1^. Although visuo-tactile interactions have been widely investigated for the estimation of the different properties of an object including its texture^2^, far less is known about the influence of auditory cues on texture perception. It has been shown that the friction noise of fingers on a surface provides valuable information about the roughness of that surface^3^. This is also demonstrated with the parchment-skin illusion paradigms, where a distortion of the auditory feedback modifies the perception of the roughness or softness of the hands rubbing against each other^4,5^. More recently, Suzuki et al.^6^ reported that a complex sound (white noise) can have a deleterious effect on the haptic discrimination task of different rough textures compared to a pure tone sound. Naturally, touch much more than hearing is a privileged sensory source to mediate texture information. The high density of cutaneous mechanoreceptors at the pulp of the fingers can accurately encode the spatial variations of the surface and also the vibrations elicited by the finger movement on the surface, which are the two main cues used for fine texture perception^7^. However, touch is affected by aging at multiple levels, from the peripheral receptors^8–10^ up to central cortical processing^11,12^. Both central and peripheral factors could contribute to age-related deficits in tactile stimuli discrimination and detection^13–18^. Strikingly, most of the studies about age-related changes in tactile perception have been restricted to static touch investigation, while there is evidence that dynamic touch is affected differently with aging^19–21^. In addition, very few studies have focused on textures perception and have shown an impairment in fine textures discrimination by varying micro-structure surfaces^22^ but no impairment when the surface grooves varied more coarsely^13,22–24^.

In complement to the degradation of each sensory system, changes in multisensory perception with age have attracted great interest in recent years^25^. Previous studies have stated that enhanced multisensory integration in the presence of congruent stimuli may be a compensatory phenomenon against age-related unisensory declines^26–31^. However, older adults are also more disturbed^32^ and more susceptible to be blurred by task-irrelevant stimuli^29,33^. For example, the erroneous perception of the number of visual flashes due to the simultaneous presentation of a different number of auditory beeps, called sound-induced flash illusion, is more easily experienced by older people^29^. This age-related difference could be related to changes in attentional processing or in sensory integrative mechanism in older people. Interestingly, using an audio-visual attention paradigm, Hugenschmidt et al.^27^ showed that the ability to engage cross-modal selective attention is preserved with aging and proposed that increase multisensory integration is not due to a failure of attention but probably to an enhancement of multisensory integrative processing.

In the present study, we examined the impact of textured sound on haptic exploration of textures, and in particular age-related changes that have been less investigated, probably due to the technical challenges inherent to the combination of textures and sounds. To address these questions, we developed an innovative approach allowing the simultaneous fine modulation of tactile and auditory textured stimuli by combining a texture simulation device (StimTac) with a sound synthesizer based on perceptually relevant acoustic morphologies^34–36^. We studied two textured sounds were used to evoke different friction levels, like a feeling of rubbing or squeaking, and one neutral sound (pure sound) were delivered during a haptic exploration task. The sounds were synthesized in real time and modulated by the finger movement velocity, but were distracting regarding the tactile discrimination task as they did not systematically correspond to the texture explored haptically. If sounds influenced haptic perception of textures, then one could expect their effect to be greater if the auditory stimulus shared some properties with the explored texture. More precisely, in the psychophysical task performed here, we predicted a gradual effect of sounds according to the increase of the evoked friction level on the tactile discrimination threshold (i.e., just noticeable difference, JND) and on the perception of the tactile stimulus judged equal to the standard stimulus (i.e., point of subjective equality, PSE). By contrast, if sounds had only a distractive effect, without being integrated with tactile information, they may deteriorate tactile discrimination performances (JND), but not tactile judgements (PSE), per se. Finally, as a facilitation of multisensory integration as well as a greater impact of sensory distraction were observed with aging, we expected that the older adults tested here should be more affected by the presence of the two textured sounds than the younger adults.

## Results

### Influence of textured sounds on haptic roughness discrimination

**Figure 1:**
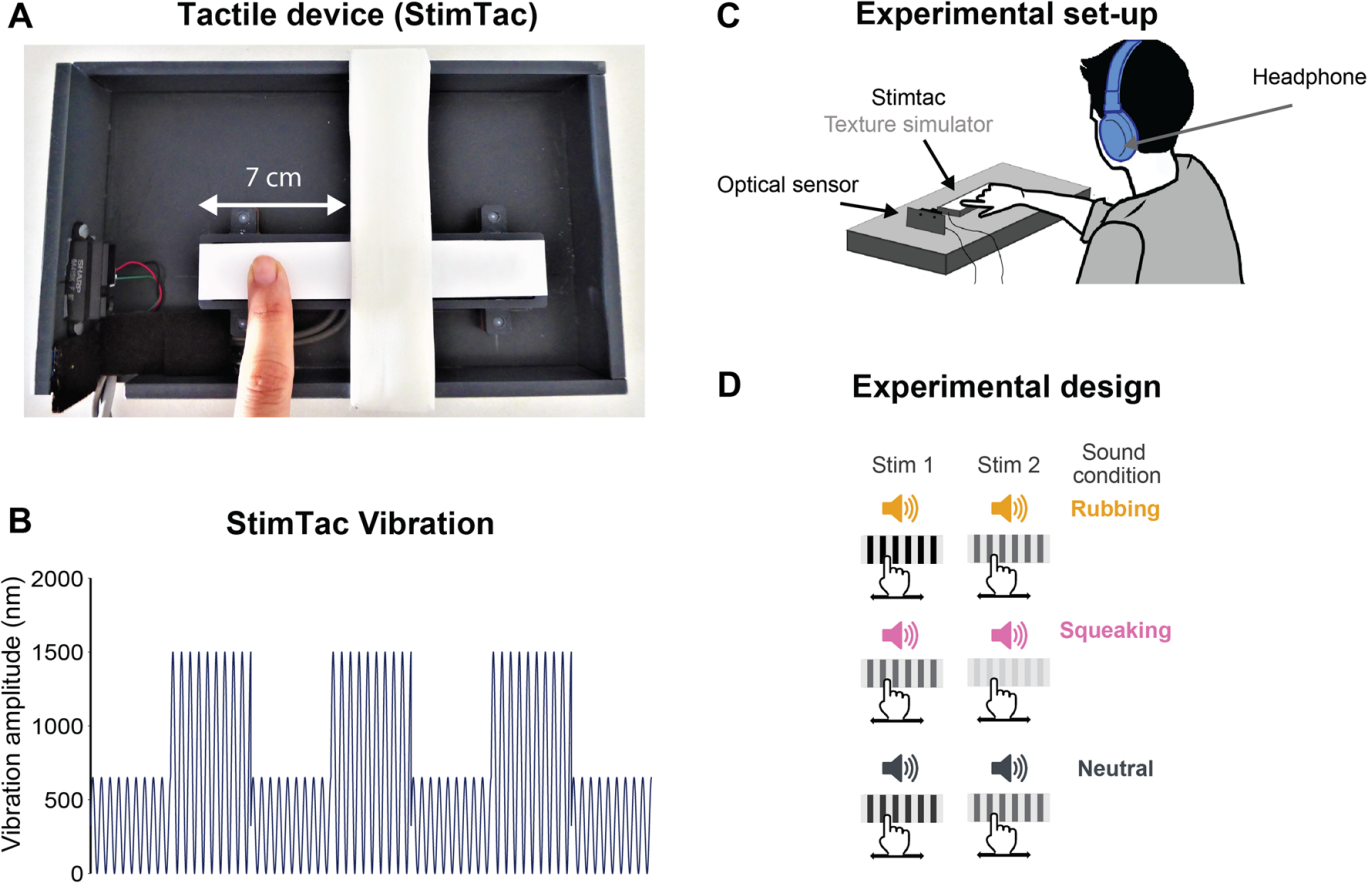
(**A**) Picture of the tactile device called ‘StimTac’. (**B**) Illustration of the StimTac’s vibration during the reference stimulation ΔAref = 750 nm consisting of a switching between two amplitudes of vibration: the highest amplitude (1500 nm) and an intermediate (750 nm). The two amplitudes were delivered alternately at 22 Hz. (**C**) Experimental set-up: seated participant explored the touchpad of the StimTac with their right index and wore headphones with synthesized sounds. An optical sensor recorded online the participant’s index displacements. (**D**) Experimental design: participants compared two different textures in presence of the same sound (that could be Rubbing, Squeaking or Neutral). During the experiment, all sound conditions were randomly presented.

To test the influence of textured sounds on haptic roughness perception, all participants explored different textures in presence of a distractive sound. Virtual textures were simulated using a device called “StimTac” consisting in a touchpad that supports friction modulation (Fig. 1A). To create a grooved sensation under the participants’ finger, ultrasonic vibrations alternated at two different amplitudes (Fig. 1B). The active exploration of the textures was always associated with a sound, which was modulated by the actual velocity of the participant’s finger movement (Fig. 1C). The sound was either a textured sound eliciting a feeling of friction (called Rubbing or Squeaking) or a pure sound (called Neutral) used as a control condition^34,35^ (Fig. 1D). The sounds were generated by a synthetizer^37^ coupled in-line to an optical sensor that recorded the actual displacements of the participants’ finger to modulate the sound accordingly (Fig. 1C).

### Discrimination thresholds

Participants underwent a two-alternative forced choice task (2AFC) to discriminate the roughness texture among a series of 6 pairs of tactile stimuli, while the sound remained identical. A seventh pair was composed of two identical reference textures. Each pair always included the reference texture (ΔA = 750 nm) and both textures were associated with the same sound, which could be the Neutral, Rubbing or Squeaking sounds (Fig. 1D). As older participants were expected to perform less well than younger ones, we adapted the protocol among three possibilities (large, medium or small range) for each participant according to their performance during the training session. This procedure ensures an accurate assessment of individual discrimination thresholds and control for the inter-individual perceptual load, i.e. the perceptual load to perform the discrimination task was similar between the two groups. As expected, older participants mostly took part in the large range protocol (8/19). Only four participants underwent the small range protocol in both groups and most of the young participants took part in the medium range protocol (15/20).

**Figure 2:**
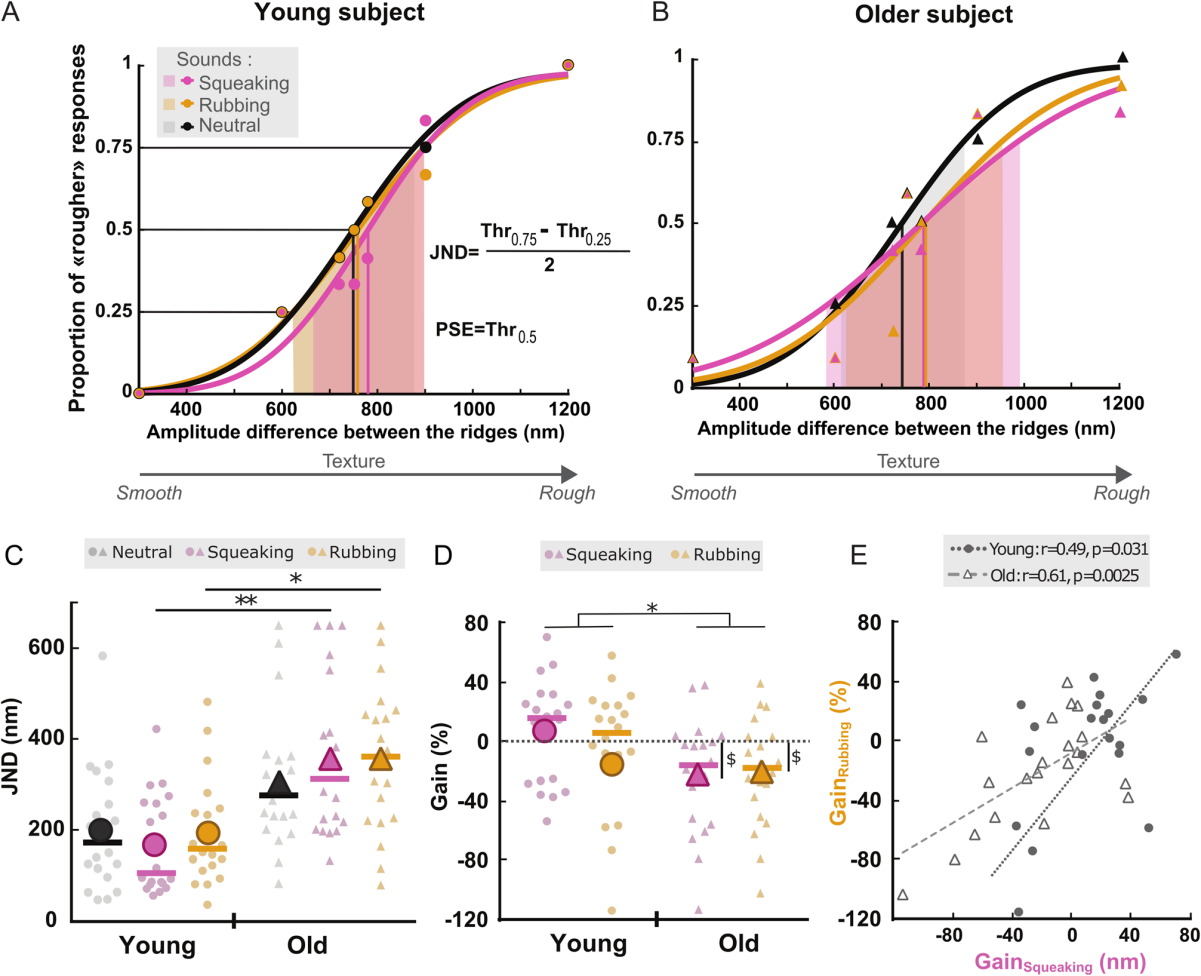
Typical psychophysical curves from one young (**A**) and one older participant (**B**) reflecting the percentage of texture stimuli perceived rougher than the reference texture (ΔA = 750 nm) for each sound condition: Squeaking (pink), Rubbing (yellow) and Neutral (black). Symbols are the mean values obtained for 7 textures varying in roughness. JND: just noticeable difference; PSE: point of subjective equality. (**C**) Individual and mean JND values for the younger (dots) and older participants (triangles) in each sound condition: Squeaking (pink), Rubbing (yellow) and Neutral (black). Statistics are post hoc tests after Holm correction * (**D**) Individual and mean Gain indexes (%) (Squeaking in pink and Rubbing in yellow) for the younger (dots) and older (triangles) participants. The largest symbols are the mean of the groups and the horizontal bars are the median. Statistics are the main effect of the GLM (*) and *t*-tests different from zero ($). (**E**) Correlations between the two Gain indexes (Squeaking and Rubbing) within each group (Young: black dot line; Old: grey dash line). * or $ *p* < 0.05; **p < 0.01. *Thr* Threshold.

To determine the proportion of textures perceived rougher than the reference one, individual psychometric functions were computed based on participants’ answers. Figure 2A,B illustrates typical individual psychometric curves for two representative young and old participants in the three sound conditions. We compared the discriminative performance by extracting the JND from all the curves. Statistical results from generalized linear mixed-effects models with a gamma link function (G_z_LMMs) analyses are reported in Table 1A. They revealed significant main effects of Group and Sound as well as significant interaction between Sound and Group (Fig. 2C). Post-hoc comparisons revealed significant differences between Younger and Older groups in the Squeaking (z = − 3.5; adjusted-*p* = 0.0041) and Rubbing (z = − 2.76; adjusted-*p* = 0.046) conditions but not in the Neutral condition (z = − 2.08; adjusted-*p* = 0.16). In other words, the discriminative performance was significantly altered in the older group compared to younger group only when they were exposed to a textured sound and not in the case of a neutral sound. No significant difference was observed between the three sound conditions within each group (Supplementary Table S1A).

**Table 1:** Summary of the statistical results from the generalized linear mixed models for the JND (A) and PSE (B) variables and the general linear mixed model for the Gain indexes variable (C). Marginal (*Rm*^2^) and conditional (*Rc*^2^) variances explained, degree of freedom (df), chi-square (Chisq) and p-value (*p*) are given.

	A: JND (nm) Rm^2^ = 0.31; Rc^2^ = 0.74			B: JND Gain indexes (%) Rm^2^ = 0.10; Rc^2^ = 0.60			C: PSE (nm) Rm^2^ = 0.13; Rc^2^ = 0.48		
	df	Chisq	*p*	df	Chisq	*p*	df	Chisq	*p*
Group	1	4.34	0.037*	1	6.14	0.013*	1	0.065	0.79
Sound	2	6.60	0.036*	1	1.98	0.16	2	0.27	0.87
Group × sound	2	9.31	0.0095**	1	1.07	0.30	2	8.72	0.013*

To further investigate to what extent textured sound distractors had a negative effect on haptic discrimination performance compared to the Neutral sound distractor, individual Gain indexes of the discrimination thresholds were computed for each textured sound condition (Fig. 2D). First, we compared the Gain indexes between the Sound and Group factors using a linear mixed-effects model (LMM). It revealed a significant main effect of Group (Chisq(1) = 6.14, p = 0.013) but no significant effect of Sound nor interaction between Sound and Group (Table 1B). The Gain indexes were found significantly lower in the older group than in the younger group. In addition, Student’s *t*-test used for the comparisons to zero showed that Gains indexes were significantly negative for the Squeaking and Rubbing sound conditions in the older group (Gain_Squeaking_: − 23.50 ± 39.1, t(18) = − 2.62, *p* = 0.017; Gain_Rubbing_: − 23.01 ± 35.6, t(18) = − 2.82, *p* = 0.011). No significant differences were found in the younger group (Gain_Squeaking_: 7.01 ± 34.6, t(19) = 0.90, *p* = 0.38; Gain_Rubbing_: − 2.95 ± 43.4, t(18) = − 0.29, *p* = 0.77) (Fig. 2D). This result reflected an impairment in haptic discrimination in presence of irrelevant textured sounds over the Neutral sound condition only in the older group.

To investigate whether the two textured sounds had opposite or identical effects on the same participant, a correlation between the two Gain indexes was computed. The analysis revealed a positive correlation within the two groups (Young: r = 0.49, t(17) = 2.35, *p* = 0.031; Old: r = 0.61, t(17) = 3.20, *p* = 0.0052) (Fig. 2E) indicating that the two sounds had a similar effect for the same participant.

**Figure 3:**
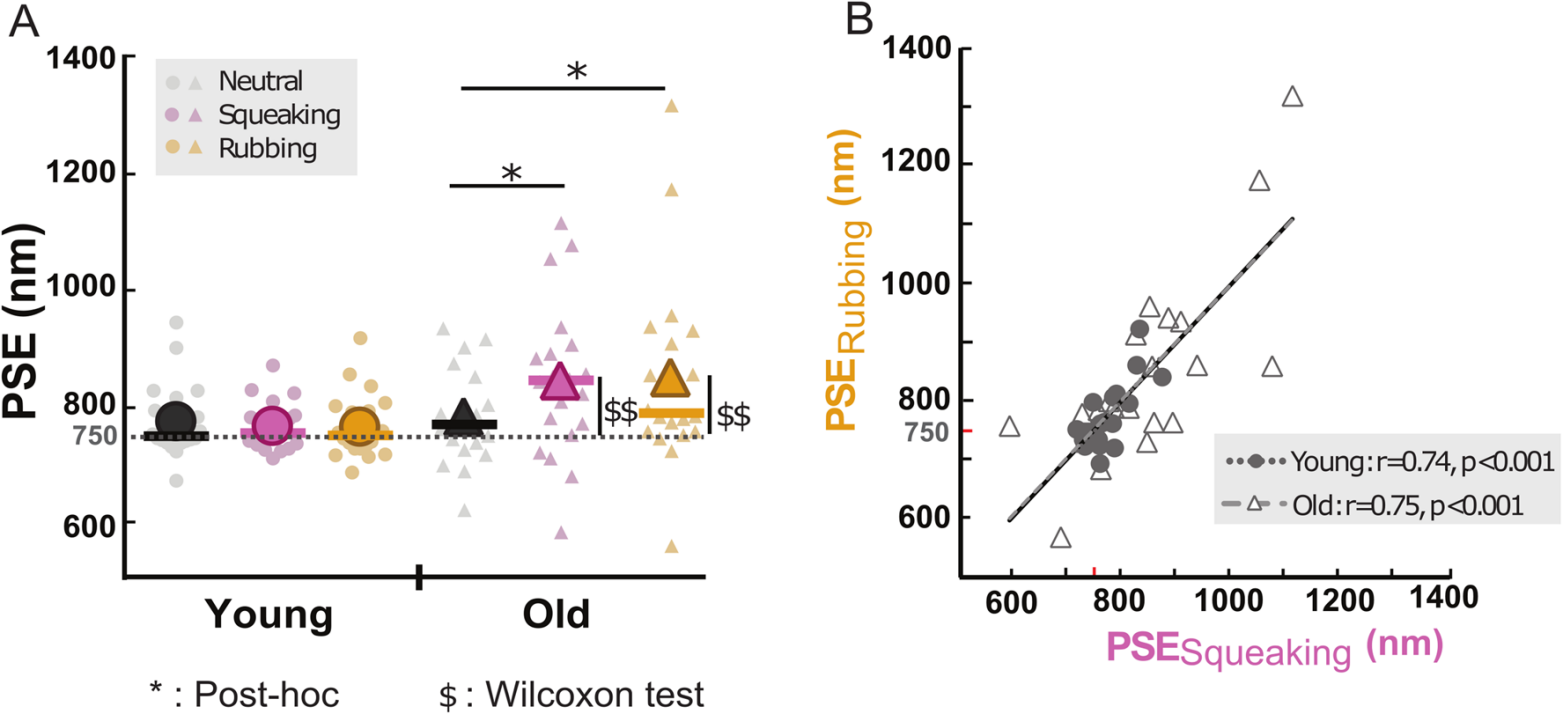
(**A**) Individual and mean point of subjective equality (PSE) values for the younger (dot symbols) and older participants (triangle symbols) in the Squeaking (pink), Rubbing (yellow) and Neutral (black) sound conditions. *p < 0.05, **p < 0.01 for post hoc tests with Holm correction; ^$^p < 0.05, ^$$^p < 0.01 for Wilcoxon tests different from zero. (**B**) Correlations between the PSE observed in the Squeaking and in the Rubbing conditions across the two groups (full line) and within each group (Young: black dot line; Old: grey dash line). Symbols are individual percentage of rougher responses compared to the reference texture for young (full dot symbols) and older (empty triangle symbols) participants.

Figure 3A showed the individual and mean PSE (e.g. the point at which participants judged 50% of the trials to be rougher than the reference) extracted from all the psychometric curves computed in the young and old groups. G_z_LMM analysis performed on PSE values revealed no significant main effects of the two factors Group and Sound, but a significant interaction (Table 1C). Post-hoc comparisons revealed significant differences in the older group between the Neutral condition and the Squeaking condition (z = − 3.11, adjusted-*p* = 0.016) as well as between the Neutral condition and the Rubbing condition (z = − 3.15, adjusted-*p* = 0.016). In other words, the older participants perceived the reference texture as being rougher in presence of a textured sound (Squeaking or Rubbing) compared to the Neutral sound. By contrast, PSE values in the younger group were not significantly different between the three sound conditions and remained close to the reference amplitude at 750 nm (Supplementary Table S1B). Post-hoc comparisons between groups for each sound condition did not reveal any significant differences.

To further investigate to what extent textured sound distractors shifted the texture perception, PSE values were statistically compared to 750 nm, which was the difference in amplitude of the texture reference value. Wilcoxon tests showed that the PSE_Squeaking_ and PSE_Rubbing_ were significantly higher than 750 nm only in the older group (PSE_Squeaking_: 853.82 ± 131.7, V = 166, *p* = 0.0028; PSE_Rubbing_: 857.51 ± 165.8, V = 171, *p* = 0.0012) and not in the younger group. For both groups, the PSE obtained in the Neutral condition did not significantly differ from 750 nm (Table 2).

**Table 2:** Comparisons of the PSE values with the reference texture value (750 nm) using Wilcoxon tests for the three texture conditions in the Young and Old groups. *p < 0.05; **p < 0.01.

Conditions	Young			Old		
	Mean ± SD	V	*p*	Mean ± SD	V	*p*
Neutral	777.40 ± 62.9	140	0.20	787.01 ±81.7	139	0.080
Squeaking	769.46 ± 40.8	150	0.097	853.82 ± 131.7	166	0.0028**
Rubbing	768.54 ± 55.8	128	0.41	857.51 ± 165.8	171	0.0012**

A correlation analysis between the PSE values in the Squeaking and Rubbing sound conditions showed a significant and strong positive correlation within each group (Young: r = 0.74, t(18) = 4.67, *p* < 0.001; Old: r = 0.75, t(17) = 4.63, *p* < 0.001) (Fig. 3B). As for the Gain indexes, the PSE varied similarly in the Squeaking and Rubbing conditions in all the participants.

### Finger movement velocity

**Figure 4:**
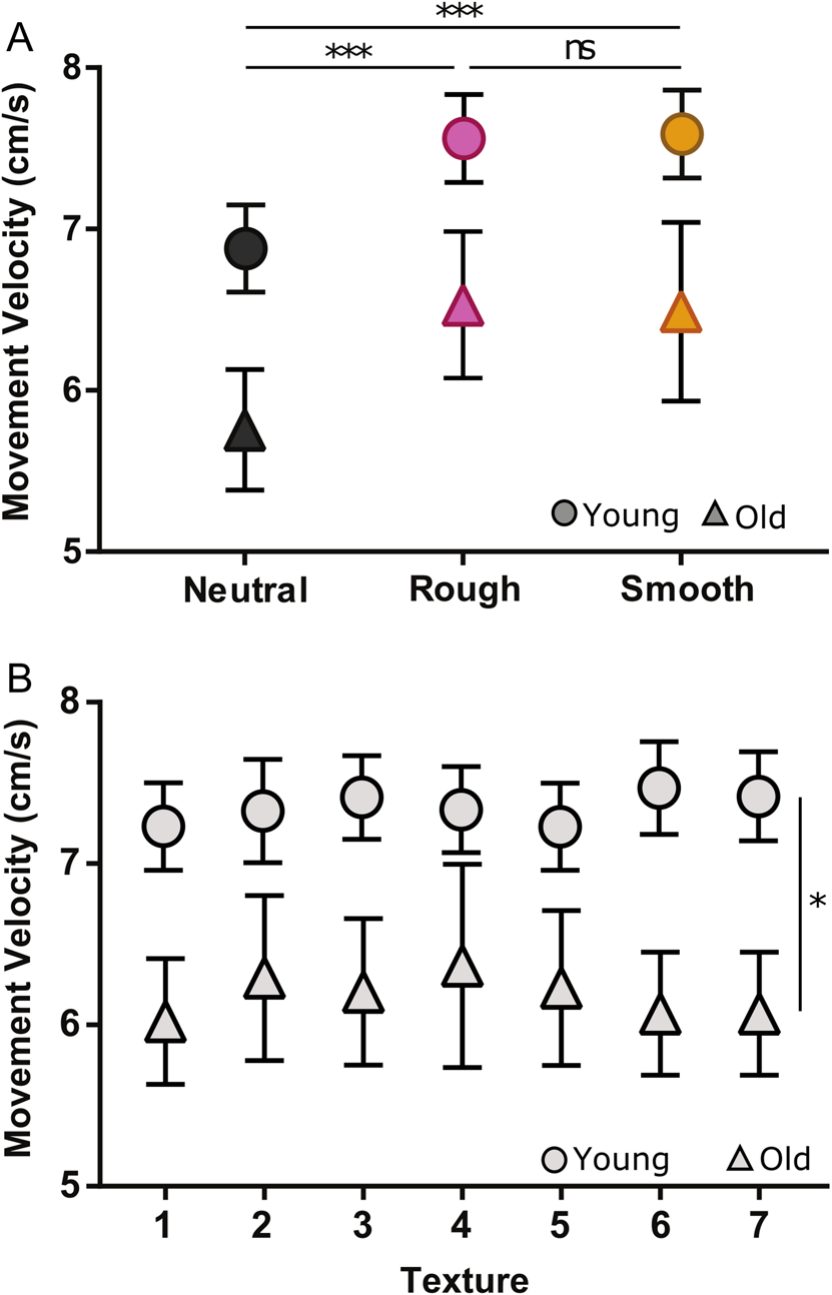
(**A**) Mean finger movement velocity (cm/s) for younger (dot symbols) and older adults (triangle symbols) for each sound condition: Squeaking (pink), Rubbing (yellow) and Neutral (black). (**B**) Mean finger movement velocity (cm/s) for younger (dot symbols) and older adults (triangle symbols) depending on the various roughness textures explored by the participants. The statistics presented are the effects of the Sound conditions for the two groups confounded (**A**) and the main effect of the Groups for all the textures confounded (**B**). *ns* non-significant, *p < 0.05; ***p < 0.001.

Finger movement velocity of the participants was compared between the groups depending on the sound and texture conditions. A linear mixed-effects model (LMM) analysis revealed significant main effect of Sound and main effect of Group but no significant main effect of Texture nor any significant interactions (Table 3). In other words, participants moved differently according to the sound condition but not to the texture (Fig. 4A,B). Post-hoc analyses among the three sounds revealed that velocity was significantly higher when Squeaking (7.03 ± 2.2 vs 6.03 ± 1.6; t = 8.64, adjusted-*p* < 0.001) and Rubbing (6.95 ± 1.84 vs 6.03 ± 1.6; t = 7.74, adjusted-*p* < 0.001) sounds were presented compared to the Neutral sound. There was no difference in finger movement velocity between Squeaking and Rubbing sounds (t = 0.90, adjusted-*p* = 0.37). Moreover, younger adults moved always faster than older adults in all sound conditions (Main effect Group: Chisq = 3.94; *p* = 0.046).

**Table 3:** Summary of the linear mixed model results for finger movement velocity. Marginal (*Rm*^2^) and conditional (*Rc*^2^) variances explained, degree of freedom (df), chi-square (Chisq) and p-value (*p*) are given.

	Velocity *Rm*^2^ = 0.12;* Rc*^2^ = 0.76		
	df	Chisq	*p*
Group	1	3.94	0.046*
Sound	2	90.34	< 0.001***
Texture	6	3.50	0.74
Group × sound	2	0.33	0.84
Group × texture	6	5.35	0.50
Sound × texture	12	14.56	0.26
Group × sound × texture	12	8.39	0.75

Finally, we also examined whether finger movement velocity was related to the JND and PSE of the two groups. First, we verified that the present results did not change when the finger movement velocity was included as a covariable into the statistical model (see Supplementary information). Secondly, we also performed correlation analyses and found that, except for the JND during the Rubbing condition in the younger group, no significant correlation was found with the movement velocity in either group under any sound conditions. In the Rubbing condition, the faster the movement of the finger, the higher the discrimination performance in young adults (Supplementary Table S2).

At the end of the experiment, the experimenter asked the participant to name the different sounds. All participants classified the Neutral sound as coming from a “motor device” or as a “cycling sound”, the Squeaking sound as a “squeaking sound”, and the Rubbing sound as a “fluid sound like the wind or waves”. When participants were asked to match the textured sounds with the virtual textures, 100% of the participants perceived the Squeaking sound more consistent with a rougher texture (ΔA = 205.13 ± 180.2 nm) and all but one perceived the Rubbing sound more consistent with a smoother texture (ΔA = 1194.87 ± 306.7 nm).

## Discussion

This study aimed to explore how sounds influenced haptic perception of textures and if aging had an effect on audio-tactile integration. In particular, we compared the effect of two textured sounds (Squeaking or Rubbing) that share some properties with the explored texture and a neural sound on tactile discrimination performance of young and old adults. The results revealed that older adults had lower tactile discrimination performance in the presence of the two textured sounds compared to the neutral one, while it was not the case in younger adults.

At first glance, one may argue that the lower discrimination performance may be explained by a non-specific attentional effect, the auditory stimuli capturing more attention in older compared to younger people^38^. However, the performance of the older group was not affected in the Neutral condition suggesting that it was not only the presence of a sound distractor that could explain the present results. Also, older people are known to be able to engage selective attention to a given sensory modality as young adults^27^. Nevertheless, because the textured sounds were more complex than the neutral sound, they may have had a different impact on the attentional effect. The finding that the PSE also deviated towards rougher textures in the presence of the Squeak and Rubbing sounds showed that the deleterious effect would be more related to the textured nature of the sound distractor. If the complex textured sounds had only an attentional effect, then the PSE should have been randomly shifted in either side of the referential texture. Taken together, these findings suggest that the impairment in roughness discrimination in the older group depends on the nature of the presented sounds. Nevertheless, we did not observe an expected gradual effect between the two textured sounds (according to the increase of the evoked friction level between the Rubbing and Squeaking sounds). To confirm this hypothesis, future studies should thus be conducted to compare the effect of textured sounds with respect to other kinds of complex sounds that do not share friction properties similar to those of haptic surface exploration.

Alternatively, the present findings might be explained by an irrelevant audio-tactile binding in older adults. This hypothesis is consistent with a vast amount of studies showing that although sensory systems deteriorate with aging, older people appear to benefit from enhanced multisensory integration to improve their perception^20,26–31^ and action^39^. In particular, multisensory integration of audio-tactile information was enhanced with aging in the context of motor performance such as finger tapping synchronisation or reaction times^40,41^. It is also often found that older adults are more disturbed when they are facing with a stimulus that is not relevant to the task^29,33^. In the present study, we manipulated the nature of the sounds in a fine texture discrimination task. The findings suggest that sound distractors with friction properties (or at least those more complex than pure sound) may further interfere with the haptic task and increase the difficulty of older people to segregate and appropriately-weight disturbing environmental cues. This is consistent with previous studies showing that if the integrated information is useful for the task, older people will likely experience a greater benefit than younger people^26–31^, but if the integrated information is not relevant for the task, as in this experiment, their performance will be deteriorated^32^. To further test this hypothesis, future studies should be conducted to determine whether adding relevant texture sounds during haptic exploration would be more beneficial to older, than younger, adults.

In contrast, young adults were able to segregate irrelevant auditory information to preserve their haptic discrimination performance. In other words, when the same sound was associated with a pair of two different textures, young participants did not use this distractive information to distinguish the relative roughness of the textures. This finding is consistent with previous observations, that providing young adults with the actual sound produced by the active touch does not improve the level of roughness perception of grating surfaces^42^, except if haptic exploration is indirectly made using a rigid probe^43^ or if the auditory feedback is amplified^44^. It is also consistent with previous results on visuo-tactile interactions showing that haptic distractors can affect visual discrimination of texture, but this effect is not symmetric^45^. Taken together, all these findings are in line with the “*Modality appropriateness*” principle according to which sensory feedback signals should be weighted depending on its behavioural relevance in a given context^46^. Given the neurophysiological properties of cutaneous mechanoreceptors, hand touch may provide more relevant information related to the texture than hearing or vision so that the brain will rely more on touch to distinguish roughness of surfaces. The finding that young participants seemed to have successfully completed the task by ignoring the textured sounds can also be explained by the fact that, as the sound was not changing with the various textures explored, auditory and tactile information may have been interpreted as emerging from different origins. In addition, the prior instruction to focus on texture discrimination could also have down weight the auditory information with respect to the tactile one, facilitating ultimately a segregation mechanism^47^. Indeed, basing the judgement on only one modality and ignoring the others is known as an extreme case of the causal inference process^48^.

Interestingly, the fact that textured sounds influenced the haptic exploration by increasing the movement velocity of the finger over the surface in all the participants but not the Neutral sound suggested that the textured sounds were implicitly taken into account during the motor execution of the haptic exploration. This is consistent with the observation that adding an auditory feedback during motor execution can improve motor skills such as rhythmic fine motor learning^49^ or learning to write^50^. In addition, movement sonification seems to have a beneficial impact on deafferented patients without somatosensory afferents^51^. However, in the present study, movement sonification per se could not fully account for the change in motor exploration: finger movements were more influenced by textured sounds than neutral sounds although the three sounds were all modulated by the movement velocity of the finger. The nature of the present sounds, and their generated friction properties similar to those of haptic surface exploration, would be responsible for this difference in motor behavior. This audio-tactile integration could have strengthened the feeling that it was the active finger touch that produced the textured sound resulting in a stronger audio-motor association. Another possibility that cannot be ruled out is a differential emotional impact of the sounds, which could have modulated their integration. Even if we never asked the participants to pay attention to the sound, all participants were able to classify the Squeaking and Rubbing sounds as squeaky and flowing sounds, respectively, and never mentioned any emotional associations related to these sounds. Nevertheless, this emotional influence of the sound should be further considered in future studies.

Finally, one may argue that the increased movement velocity during the haptic exploration in presence of the textured sounds with respect to the Neutral sound may have affected the older group but not the younger group, which would have better overall performance. However, the fact that increasing the velocity of finger displacement tended to be negatively correlated with the discriminative thresholds in the three sound conditions and in both groups does not support this hypothesis. Furthermore, when it was significant, as in the Rubbing condition, the increase in finger movement velocity coincided with better texture discrimination performance in young participants. Lastly, by including the movement finger as a covariate variable in the main model of the statistical analysis, we verified that the present results were not changed. Altogether, these arguments led us to conclude that the movement of the finger did not seem to interact with the perception of the texture during the discrimination task (JND or PSE).

## Conclusion

Using an innovative multisensory set-up based on synthesized audio-haptic stimuli, the study showed that movement sonification influences the active exploration of a textured surface, especially when the sound contains frictional information. Nevertheless, audio-haptic interactions seem complex and affected unequally the young and old adults in a roughness discrimination task. When textured sounds were not relevant for haptic roughness tasks, younger adults were unaffected by their presence. In contrast, older adults were not able to segregate irrelevant sounds. These results support the hypothesis of a general facilitation of multisensory integration processes in the elderly, which may be advantageous or disruptive depending on the context.

## Methods

### Participants

Twenty older (mean 67.4 ± 2.5 years; 9 men) and twenty younger volunteers (mean 22.6 ± 2.2 years; 8 men) participated in the study. All participants were right-handed, according to the Edinburgh handedness scale^52^. They reported to have normal hearing and had no skin disease or any history of neurological or sensorimotor diseases. A Mini-Mental State score above 26 and a preserved daily life autonomy were required for older participants.

### Statement of human rights

All procedures performed in the present study were in accordance with the ethical standards of the institutional and/or national research committee (approved by the Comité de protection des personnes CPP Ouest II Angers N° 2018-A02607-48) and with the 1964 Helsinki declaration and its later amendments or comparable ethical standards. All participants gave written informed consent.

### Apparatus and stimuli

#### Tactile stimuli

The “stimTac” device simulated virtual textures in a touchpad device of 12 × 2.3 cm that supports friction modulation (Fig. 1). It is driven by a controlled vibration at an ultrasonic frequency with a few micrometers amplitude to create an air gap (squeeze film) that spreads between the user’s finger and the whole device’s surface (more details in Supplementary information). To create a grooved sensation under the participant’s finger, two vibration amplitudes alternated at a frequency of 22 Hz. One of these two amplitudes corresponded to the maximal amplitude of 1500 nm, i.e. corresponding to a very smooth sensation. The second amplitude, corresponding to a rougher sensation, varied between 1300 and 200 nm. Therefore, the difference between the two amplitudes (ΔA) determined the texture simulation: the higher the difference, the rougher the perceived groove. The intermediate delta amplitude of vibration ΔAref = 750 nm was the reference texture used in the discrimination task.

#### Auditory stimuli

Sonification was generated in real time with Max software (http://cycling74.com). In the Neutral (control) condition, a pure sound was associated with the movement velocity. In the two textured (experimental) conditions, the synthetized friction sounds resulted from the *action-object* paradigm^34,35^. This paradigm describes the sound as the result of an action on an object and presumes the existence of sound invariants (i.e., perceptually relevant signal morphologies that carry information about the action’s and/or the object’s attributes). The notion of sound invariants was first adapted from vision to auditory perception by Warren and Verbrugge^53^ and formalized by McAdams and Bigand^54^ who split auditory sound invariants in two categories: *Structural invariants* responsible for the recognition of physical properties of a sounding object (its material, shape, etc.) and *transformational invariants* responsible for the recognition of the action on the object (breaking, rolling, etc.). Thanks to this categorical distinction, it is possible to recognize a bottle by the sound it produces, and whether it bounces or breaks. In the current study the two textured sounds were obtained from the evocation of two different actions, namely rubbing and squeaking. These actions were produced on the same object (a plastic surface) to determine if differences in the action attributes may affect the perceived roughness, with a squeaking sound evoking greater roughness than a rubbing sound. More details including the signal spectrum of the three sounds are provided in Supplementary information^55^ and a video is available with the electronic version of the manuscript.

#### Movement caption and sonification

An optical sensor was used to record the displacements of the participant’s finger on the touchpad (Fig. 1A). Finger movements were recorded at a sampling rate of 200 Hz, filtered with a fourth order low pass butterworth filter (6 Hz), and derived to extract the instantaneous movement velocity in real time required by the synthesizer for modulating the sound accordingly.

### Procedure

#### Training phase

All the participants were trained to move their finger back and forth along the 7 cm distance of the touchpad with their index finger in 2.7 s (stimulation duration), i.e. at a predefined 5.2 cm/s velocity (based on pilot tests carried out on 10 young participants using the same textures, for more details see Supplementary information).

Then, participants were trained to the tactile discrimination task with their eyes closed. The seven textures used in this training phase were the same for all participants: ΔA1 = 1300 nm; ΔA2 = 950 nm; ΔA3 = 800 nm; ΔA4 = 750 nm; ΔA5 = 700 nm; ΔA6 = 550 nm; ΔA7 = 200 nm. All participants but one in the older group were able to perform the task accurately in the training phase. Therefore, 20 young adults and 19 older adults were included in the whole experiment (more details in Supplementary information).

#### Testing phase

Participants were exposed to pairs of audio-haptic stimulations and underwent a 2AFC discrimination task with always the same instruction: “Which was the roughest texture you explored between the two?”.

Pairs of two different textures were presented with the same sound: Neutral, Squeaking or Rubbing (Fig. 1D). According to their individual performance during the training session, the seven textures were adapted to each participant among three protocols: ‘large range’, ‘medium range’ or ‘small range’. (large range: ΔA1 = 1400 nm; ΔA2 = 1000 nm; ΔA3 = 850 nm; ΔA4 = 750 nm; ΔA5 = 650 nm; ΔA6 = 500 nm; ΔA7 = 100 nm, medium range: ΔA1 = 1300 nm; ΔA2 = 950 nm; ΔA3 = 800 nm; ΔA4 = 750 nm; ΔA5 = 700 nm; ΔA6 = 550 nm; ΔA7 = 200 nm, small range: ΔA1 = 1300 nm; ΔA2 = 900 nm; ΔA3 = 780 nm; ΔA4 = 750 nm; ΔA5 = 720 nm; ΔA6 = 600 nm; ΔA7 = 200 nm). This setting allowed us to obtain precise estimates of the discrimination threshold of each participant^21^ and to ensure that the perceptual load of the task was similar across the participants. Indeed, comparing textures with a large difference in amplitude may have been too easy for a young participant, while comparing textures with very small differences in amplitude may have been too difficult for an older adult. The same Neutral, Rubbing and Squeaking sounds were delivered to all participants, regardless of the three protocols.

A total of 252 trials were tested, including 12 repetitions × seven pairs of textures × three sound conditions, randomly interspersed over 12 sessions. All the trials lasted for 8.2 s (including the two 2.7 s stimulation duration, 0.5 s of rest between the stimulation and 2.3 s of rest to answer).

For all trials, participants were instructed to focus on texture discrimination despite the presence of different types of sound. The nature and number of sounds were never described by the experimenter until the end of the experiment. Thus, the participant was completely naive about auditory stimulation.

### Data and statistical analyses

Statistical analyses were carried out using R Core Team 3.6.1 programming language. To evaluate the influence of experimental variables (e.g. sounds, textures, groups), we fitted with linear mixed models^56^ (LMM) for dependent variables with normal distributions (e.g. Gain indexes and finger movement velocity) or generalized linear models (GLMMs) for response variables with non-normal distributions (e.g. JND, PSE) using the R package ‘lme4’ (version 1.1-21)^57^. These models took into account the effects of experimental variables (fixed-effects), the variability between subjects (random effects) and the residual error. They were fitted to data by the maximum likelihood method, providing estimation parameters (slope, intercepts, means) and the estimated of their standard errors (SE). Main effects and interactions were analyzed using Wald statistical tests (*Anova* function in the R package ‘*car*’). The χ^2^, degrees of freedom and *p*-values are reported in the results section for all main effects and interactions.

Significant interactions and main effects (p < 0.05) were further analyzed using post-hoc pairwise comparisons of the estimated marginal means (*emmeans* package in R, version 1.4). In particular, when interactions were significant, each level of fixed effects (e.g. Groups, Sounds or Textures) was compared within (levels of the same fixed-effect) and between (levels of different fixed-effects) fixed-effects. When the main effects were significant, but not the interaction, the same procedure was applied but only by comparing the levels within each significant fixed-effect. All post-hoc tests were corrected for multiple comparisons using Holm correction for *p*-value adjustment. Comparisons were considered significant at adjusted-*p* < 0.05. The resulting z-ratio or *t-*values respectively from GzLMM or LMM post-hoc comparisons and corresponding adjusted-*p*-values were reported.

Models were assessed for goodness-of-fit to the data using the marginal (*Rm*^2^) and conditional (*Rc*^2^) coefficient of determination^58^. *Rm*^2^ represents the variance explained by the fixed effects and *Rc*^2^ represents the variance explained by the entire model, including both fixed and random effects.

Statistical tests used for all variables are summarized in the Table 4.

**Table 4:** Summary of the statistical models used for the analysed variables.

Variables	Distribution	Factors (levels)	Statistical model
JND (nm)	Gamma	Group (Young, Old) Sound (Neutral, Squeaking, Rubbing)	GzLMM
JND Gain index (%)	Normal	Group (Young, Old) Sound (Squeaking, Rubbing)	LMM Student *t*-test Correlation
PSE (nm)	Gamma	Group (Young, Old) Sound (Neutral, Squeaking, Rubbing)	GzLMM Wilcoxon test Correlation
Movement (°/s)	Normal	Group (Young, Old) Sound (Neutral, Squeaking, Rubbing) Texture (1, 2, 3, 4, 5, 6, 7)	LMM
Von Frey (g)	Discrete	Group (Young, Old)	Mann–Whitney test

#### Discrimination performance

For each sound condition, a psychophysical curve of texture discrimination was computed based on a cumulative Gaussian function fitted to individual proportion of “rougher than the reference” response using Palamedes MATLAB toolbox. The JND and the PSE were extracted from each of the three individual psychophysical curves. JND was half ΔA difference between 75 and 25% points of the psychometric function estimated in each sound condition (JND_Neutral_, JND_Rubbing_, JND_Squeaking_). Therefore, a lower value of JND corresponded to a lower discrimination threshold and a better performance. PSE is the 50% threshold and corresponds to the difference in delta amplitude ΔA required for the participant to perceive a texture as rough as the reference texture.

JND and PSE were assimilated as positively skewed continuous response variables modelled by Gamma distribution. These response variables were analyzed using G_z_LMMs. Models included Groups (Young, Old) and Sounds (Neutral, Rubbing, Squeaking) as fixed factors and random intercepts for individual subjects (random effects term). This model took into account the fact that participants may have different “baseline perception”.

Since PSE values were not assimilated to normal variables, we compared the PSE values observed in the two textured sound conditions to the reference texture value 750 nm, using Wilcoxon tests.

The relative improvement or depression of performance in a textured sound condition compared to a neutral sound was also computed as a Gain index:

$$Gain_{Rubbing} = \frac{{JND_{Neutral} - JND_{Rubbing} }}{{JND_{Neutral} }},$$

$$Gain_{Squeaking} = \frac{{JND_{Neutral} - JND_{Squeaking} }}{{JND_{Neutral} }}.$$

Thus, a negative value of the Gain index indicated an increase in the JND parameter during a textured sound condition compared to that observed during the Neutral sound condition, corresponding to a lower discrimination performance, whereas a positive value would indicate a decrease in the JND parameter reflecting a better performance. For these two Gain indexes, normality was attested by Shapiro–Wilk normality tests (Young: *p* = 0.06; Old: *p* = 0.3). As the *p*-value was close to 0.05 in the younger group we further used visual inspection of quantile–quantile plots (qqplots) to confirm that the distributions of the Gain indexes were normal (see Supplementary Fig. S3). The Gain indexes were thus analyzed using LMMs that included Groups (Young, Old) and Sounds (Rubbing, Squeaking), as fixed factors and random intercepts for individual subjects (random effects term). Student’s *t*-tests were also applied to distinguish these variables from zero.

To investigate the extent to which the PSEs and Gain indexes varied in the Squeaking and Rubbing conditions (e.g. whether the two textured sounds had opposite or identical effects on the same participant), correlation analyses were computed.

#### Finger movement velocity

The mean velocity of the back and forth displacements of the participants’ finger during the haptic exploration of the touchpad was computed for each trial. One young and one old participant were excluded from the velocity analysis because of a technical problem during finger movement acquisition. The data normality was verified using the Shapiro–Wilk normality test and a LMM analysis was used to compare the mean movement velocity between Groups (Young, Old), Sounds (Neutral, Rubbing, Squeaking), and Textures (1–7). This model allowed us to take into account the heterogeneity of the participants’ observations because the textures tested were adjusted for each participant. LMM accounted for the variability of within-participant factors (Sound and Texture) and between-participants factor (Group) and included random intercept for each participant.

To further explore to what extent finger movement velocity influenced our psychophysical results (e.g. JND and PSE), we used a sequential approach that consisted of comparing two statistical models. We compared the first GzLMM model defined as y = Audio × Group + 1|subject to a second model that included movement as covariate and defined as y = Audio × Group + Movement + 1|subject. We used the Akaike’s information criterion (AIC) value to reveal which of the two models better fitted the present data, for both JND and PSE variables.

## Supplementary Information

Supplementary Video 1.

Supplementary Information.
