# Supplementary material for: The impact of movement sonification on haptic perception changes with aging
Source: Sci Rep. 2021 Mar 4;11:5124. doi: 10.1038/s41598-021-84581-3 (PMC7933169; doi:10.1038/s41598-021-84581-3)
Supplement: Supplementary file 2 — Supplementary Information. [file 41598_2021_84581_MOESM2_ESM.docx]

**The impact of movement sonification on haptic perception changes with aging**

*C Landelle, J Danna, B Nazarian, M Amberg, F Giraud, L Pruvost, R Kronland-Martinet, S Ystad, M Aramaki, A Kavounoudias*

**Supplementary information**

**Supplementary Methods**

**Material and experimental setup**

*Tactile Stimuli*. Virtual textures were simulated using a device called “StimTac”. The touchpad is animated by a controlled vibration at an ultrasonic frequency with a few micrometers amplitude to create an air gap (squeeze film) that spreads between the user’s finger and the whole device’s surface. As vibration frequency is in the ultrasonic frequency range (~42 KHz), it remains inaudible (since above 20 kHz) and imperceptible by the skin receptors of the human hand, which can detect vibrations below 1 kHz. As the vibration amplitude increases, the air gap under the finger increases, reducing the coefficient of friction between the surface and the skin, making the touchpad more slippery. More details about the StimTac has been provided in previous studies^2,3^.

*Auditory Stimuli*.

The three types of auditory stimuli (Rubbing, Squeaking and Neural) were generated by a synthesizer based on perceptually relevant acoustic morphologies. Previous studies on the perception of various sound categories have led to a new sound synthesis paradigm called the action/object paradigm^4,5^, which considers any sound as the consequence of an action on an object and which is based on a semantic description of the sound. In this paradigm, the action can be associated with the dynamics of a sound (temporal evolution) and the object with a sound texture^6^.

Squeaking sound had both stochastic and deterministic contents, the rubbing sound was mainly stochastic. The neutral sound control was a 100 Hz pure tone.


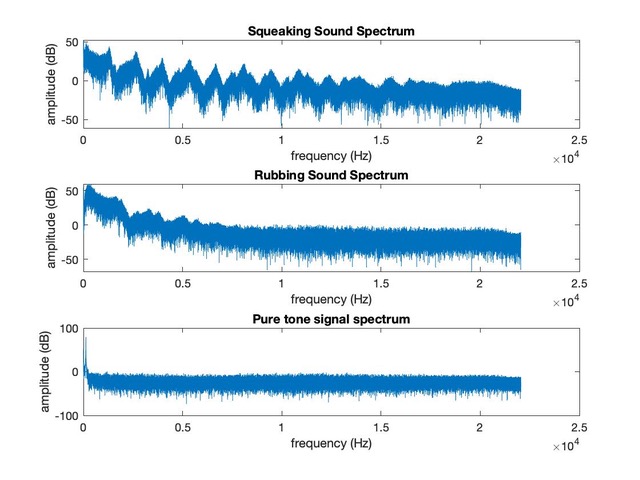


100 Hz

**Figure S1** **|Signal spectrum of the three sounds used in the present study**.

*Finger movement caption and sonification*

An optical sensor was placed 3 cm from the touchpad of the StimTac to record the displacements of the participant’s finger. Finger movements were recorded at a sampling rate of 200 Hz, filtered with a butterworth fourth order low pass filter (6 Hz), and derived to extract the instantaneous movement velocity in real time required by the synthesizer for modulating the sound accordingly. An interface for the experimenter has been designed to visualize the participants’ finger movement velocity online to control that they performed the task appropriately. The guidance of audio and haptic stimulation, as well as the finger movement acquisition were controlled by a custom program implemented in the NI LabVIEW environment (Figure S1 supplementary data).


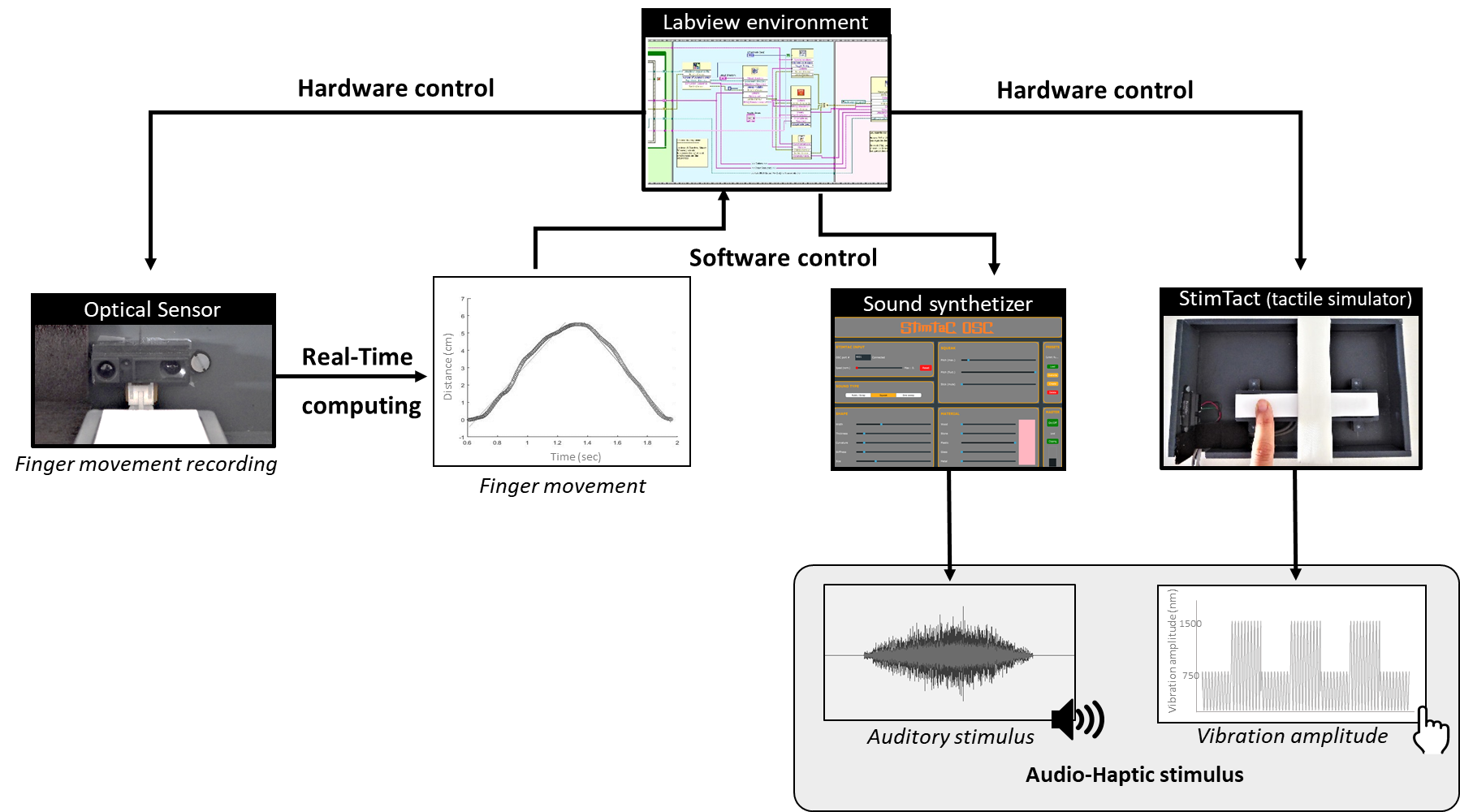


**Figure S2** **| Illustration of the stimulation protocol**. A specific software developed for the present study in the LabVIEW environment (National Instruments) controlled the guidance of audio and haptic stimulation, as well as the acquisition of the finger movements.

**Pre-experimental tests & training**

*Pre-experimental tests*

We measured the pressure threshold detection at the distal phalanx of the right index finger using von Frey monofilaments. To prevent adaptation due to repeated stimulation on the same finger, tactile pressure was exerted randomly on one of the five fingers, and participants had to locate the tactile stimulation. We used a staircase protocol starting with the monofilament 0.16 g typically perceptible by a neurologically intact population. The intensity was progressively reduced until the participant did not answer or made a mistake and we restarted the procedure 2 times. The tactile detection threshold was determined as the smallest filament perceived 3 times correctly at the distal phalanx of the index finger*.*

Thresholds of pressure detection were compared between groups using the Mann Whitney test. We also performed Pearson linear correlation between the pressure detection thresholds and the discrimination thresholds JNDs in Neutral condition within each group and across all participants.

The auditory detection threshold was also tested for all participants using the Neutral sound. The threshold was determined by the volume at which the participant detected a sound in the headphone. The auditory stimuli were then set at 10 dB above the threshold for the following experimental phase.

Participants underwent a familiarization phase during which they explored all the simulated textures used in the testing phase two times. They were also trained to always move their finger at a predefined velocity. This velocity used for the experiment was based on a pilot test carried out on 10 young participants (6 women, 24 ± 2 years). In this pilot experiment, participants were trained to move back and forth along 5 cm distance on the same tactile device as the one used in the present experiment (StimTac) at an average velocity of 4 cm/sec. All participants were able to discriminate two consecutive textures in a range of amplitude difference (ΔA) between 400 and 1000 nm (same range as the present study) with respect to a reference texture fixed at 750 nm. We were thus able to construct reliable psychophysical curves from the 10 participants. We also verified that finger movement velocity did not significantly vary as a function of the different textures explored. As we planned to test older adults with a presumed lower tactile acuity than younger adults, we slightly increased the distance exploration to 7cm (instead of 5 cm) and the movement velocity (5 cm/s instead of 4 cm/s) to have the advantage of providing more information, without increasing the total duration of the experiment too much.

Since the participants had their eyes closed, a stop was placed at a distance of 7 cm to indicate the end to be reached. A beep indicated the beginning of haptic exploration. The speed and trajectory of the finger movement were calculated online, so that the experimenter could give verbal feedback to the participant.

*Training phase*

Participants had to perform a two-alternative forced choice discrimination task with their eyes closed. Indeed, it has been shown that closing the eyes, even in complete darkness, improved somatosensory perception by switching off visual dominated processing networks^6^. In addition, this switch toward the non-visual dominated mode decreased with age^7^. Thus, we choose to carry out the present discrimination task with the participants’ eyes closed to facilitate somatosensory perception, especially among the older adults

Each test consisted of actively and successively exploring a pair of simulated textures in presence of the same Neutral sound (Fig. 1C and details in Method section). Pairs of textures always included the reference texture randomly presented in the first or second position. After two consecutive haptic explorations spaced by 0.5 s of rest, the participant had 2.3 s to report loudly which was the roughest texture between the two. Participants had to compare seven textures to the reference, randomly presented and repeated twice. The textures used in this training phase corresponded to the ‘*medium range*’ protocol (ΔA1 = 1300 nm; ΔA2 = 950 nm; ΔA3 = 800 nm; ΔA4 = 750 nm; ΔA5 = 700 nm; ΔA6 = 550 nm; ΔA7 = 200 nm). Each trial lasted 8.2 s was repeated twice leading to less than 5 minutes duration for the training phase. All the participants but one in the older group were able to perform the task accurately in the training phase. Therefore, 20 young adults and 19 older adults were included in the whole experiment.

Depending on individual performance during this training session, the seven textures were adapted to each participant among three different range protocols: ‘large range’, ‘*medium range*’ or ‘small range’. This setting allowed us to control the inter-individual perceptual load and to obtain precise estimates of the discrimination threshold of each participant ^21^. In other words, if the protocol is not adapted to the capacities of the participant, the fitting of its psychophysical curve will be less accurate or even impossible.

In the large range protocol, the delta amplitude range of the seven textures tested was the highest (ΔA1 = 1400nm; ΔA2 = 1000nm; ΔA3 = 850 nm; ΔA4 = 750nm; ΔA5 = 650nm; ΔA6 = 500nm; ΔA7 = 100nm). The delta amplitude range was reduced in the medium range protocol (ΔA1 = 1300nm; ΔA2 = 950nm; ΔA3 = 800nm; ΔA4 = 750nm; ΔA5 = 700nm; ΔA6 = 550nm; ΔA7 = 200nm) and reduced even further for the small range protocol (ΔA1 = 1300nm; ΔA2 = 900nm; ΔA3 = 780nm; ΔA4 = 750nm; ΔA5 = 720nm; ΔA6 = 600nm; ΔA7 = 200nm). The reference texture (ΔAref = 750nm) in each protocol was the same.

*Testing phase*.

In order to maintain a constant state of attention each session lasted only 5 minutes and participants took a break every 4 sessions (20 min). The full experimental testing phase lasted about 1h30.

**Data and statistical analysis**

Psychophysical and optical sensor data were processed using MATLAB R2016a.

*Finger movement velocity.* The mean velocity of the back and forth displacements of the participants’ fingers during the haptic exploration were computed for each trial. To this end, the two minimum positions (i.e. start and end positions) and the maximum position (i.e. between the back and forth) were first identified. The mean velocities of the back and forth displacements were calculated between each minimum and maximum position using linear regression. The absolute value of the two regression coefficients was then averaged to obtain the mean movement velocity along the full trial.

**Supplementary results**

**JND Gain indexes quantile-quantile plots**


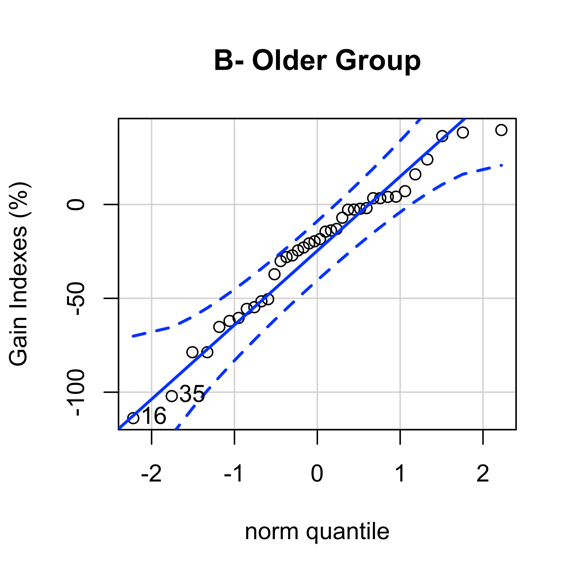

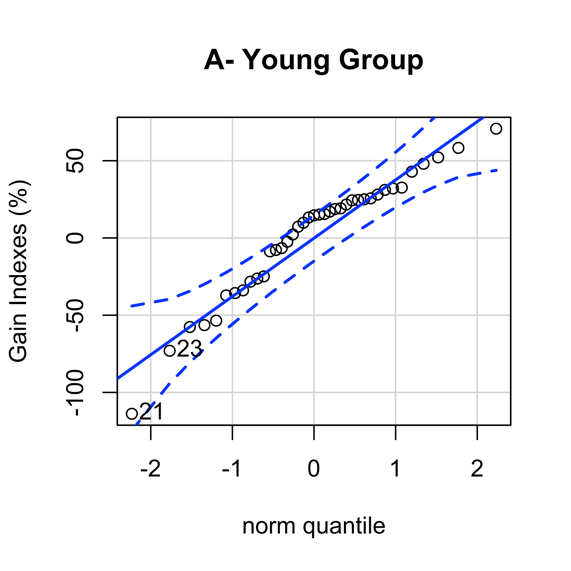


Figure S3: quantile-quantile- plots (Q–Q plots) of Gain indexes in % for Younger (A) and Older (B) groups. The linearity of the points as well as the fact that they lie within the confidence intervals suggests that the data are normally distributed.

**Post-hoc comparison on JND and PSE after GzLMMs**

**Table S1 | Post-hoc comparisons on JND values (A) or PSE values (B) depending on the groups or the different sound conditions**. The comparison names mean± standard deviation, *z-*ratio and *p-*value were reported in the table. *p*-values were adjusted with Holm method for the 8 tests. * adjusted*-p* < 0.05; **adjusted*-p* < 0.01. N=Neutral, R=Rubbing, S= Squeaking , JND= Just Noticeable Difference, PSE=Point of Subjective Equality.

|  | **A- JND** | | | **B- PSE** | | |
| --- | --- | --- | --- | --- | --- | --- |
| **Comparison** | **[mean _± SD_ ) – [mean _± SD_ ]** | **z** | ***adjusted-p*** | **[mean _± SD_] – [mean _± SD_ ]** | **z** | ***adjusted-p*** |
| [Young,N] - [Old,N] | [200.50 _± 133.4_] – [306.46 _± 148.6_] | -2.08 | .16 | [777.40 _± 62.9_] – [787.01_±81.7_] | -.25 | 1 |
| [Young,S] - [Old,S] | [68.60 _± 107_]–[357.58 _± 173.1_] | -3.51 | **.0041**** | [769.46 _± 40.8_] – [853.82 _± 131.7_] | -2.11 | .27 |
| [Young,R] - [Old,R] | [194.49 _± 116.4_] – [357.67 _±159.6_] | -2.76 | **.046*** | [768.54 _± 55.8_ ] – [857.51 _± 165.8_] | -2.18 | .24 |
| [Young,N] -[Young,S] | [200.50 _± 133.4_]–[168.60 _± 107_] | 2.12 | .16 | [777.40 _± 62.9_] – [769.46 _± 40.8_] | .40 | 1 |
| [Young,N] - [Young,R] | [200.50 _± 133.4_] – [194.49 _±116.4_] | -.18 | 1 | [777.40 _± 62.9_] – [768.54 _± 55.8_] | .49 | .99 |
| [Young,S]- [Young,R] | [168.60 _± 107_]–[194.49 _± 116.4_] | -2.30 | .15 | [769.46 _± 40.8_] – [768.54 _± 55.8_] | .089 | 1 |
| [Old,N] - [Old,S] | [306.46 _± 148.6_] – [357.58 _± 173.1_] | -2.19 | .16 | [787.01 _± 81.7_] – [853.82 _± 131.7_] | -3.11 | **.016*** |
| [Old,N] - [Old,R] | [306.46 _± 148.6_]–[357.67 _± 159.6_] | -2.22 | .16 | [787.01 _± 81.7_] – [857.51 _± 165.8_] | -3.15 | **.016*** |
| [Old,S] - [Old,R] | [357.58 _± 173.1_]–[357.67 _± 159.6_] | -.032 | .97 | [857.51 _± 165.8_] – [853.82 _± 131.7_] | -.045 | 1 |

**Finger movement supplementary analyses**

**Table S2 | Correlation analyses between finger velocity and JND or PSE for the different sound conditions in each group**. *r-, t-* and *p-*values are reported in the table. The *p*-value threshold was adjusted with Bonferroni method for multiple dependent correlation to p < 0.017 (0.05/3)

|  | | | **Correlation analyses JND** | | | **Correlation analyses PSE** | | |
| --- | --- | --- | --- | --- | --- | --- | --- | --- |
| **Group** | **Audio** | **r** | | **t** | ***p*** | ***r*** | **t** | ***p*** |
| Young | Neutral | -0.43 | | -1.97 | 0.065 | -0.26 | -1.19 | 0.25 |
|  | Squeaking | -0.35 | | -1.55 | 0.14 | 0.18 | 0.76 | 0.46 |
|  | Rubbing | -0.58 | | -2.90 | **0.010*** | -0.41 | -1.86 | 0.08 |
| Old | Neutral | -0.31 | | -1.30 | 0.21 | -0.41 | -1.83 | 0.085 |
|  | Squeaking | -0.32 | | -1.34 | 0.20 | -0.044 | -0.17 | 0.86 |
|  | Rubbing | -0.26 | | -1.08 | 0.29 | 0.077 | 0.31 | 0.75 |

To verify to what extent finger movement influenced our results, we used a sequential approach that consisted of comparing two statistical models. We compared the first GzLMM model defined as y = Audio * Group + 1|subject to a second model that included movement as covariate and defined as y = Audio * Group + Movement + 1|subject. The results of the Model 1 are the one already developed in the result section of the manuscript, showing significant interaction for both JND (Chsiq= 9.31, p=0.0095) and PSE (Chsiq= 8.72, p=0.013) variables. Model 2 showed no effect of movement (JND: Chsiq=2.64, p=0.10, and PSE: Chsiq= 0.42, p=0.51) and the interaction between Sound and Group was still significant (JND: Chsiq= 9.43, p=0.009 and PSE: Chsiq= 8.01, p=0.012). In addition, the Akaike’s information criterion (AIC) value revealed higher value for Model 1 (JND: AIC=1335, PSE: AIC=1345) than the Model 2 (JND: AIC= 1272, PSE: AIC=1283), which means that Model 1 fitted the data better than Model 2, for both JND and PSE variables. This analysis confirmed that finger movement velocity was not a covariate that influence the JND either the PSE.

**Tactile pressure detection**

Pressure detection thresholds estimated on the finger skin using Von Frey monofilaments were significantly higher in the older group compared to the younger group (Young threshold = 0.022 ± 0.006g *vs* Old = 0.11 ± 0.11g; Mann-Whitney, U = 30, *p* < 0.001). No correlation was found between the pressure detection threshold and the discrimination threshold JND_Neutral_ within each group (Young: r = 0.39, t(18) = 1.82; *p* = 0.085; Old: r = -0.081, t(17) = -0.34, *p* = 0.74) and across all participants (r = 0.12, t(37) = 0.78, *p* = 0.44).

**Supplementary references**

1. Oldfield, R. C. The assessment and analysis of handedness: the Edinburgh inventory. *Neuropsychologia* **9**, 97–113 (1971).

2. Amberg, M. *et al.* STIMTAC: a tactile input device with programmable friction. in *Proceedings of the 24th annual ACM symposium adjunct on User interface software and technology - UIST ’11 Adjunct* 7 (ACM Press, 2011). doi:10.1145/2046396.2046401.

3. Giraud, F., Amberg, M., Lemaire-Semail, B. & casiez, G. Design of a transparent tactile stimulator. in *2012 IEEE Haptics Symposium (HAPTICS)* 485–489 (IEEE, 2012). doi:10.1109/HAPTIC.2012.6183835.

4. Aramaki, M., Besson, M., Kronland-Martinet, R. & Ystad, S. Controlling the Perceived Material in an Impact Sound Synthesizer. *IEEE Trans. Audio Speech Lang. Process.* **19**, 301–314 (2011).

5. Conan, S. *et al.* An Intuitive Synthesizer of Continuous-Interaction Sounds: Rubbing, Scratching, and Rolling. *Computer Music Journal* **38**, 24–37 (2014).

6. Thoret, E., Aramaki, M., Gondre, C., Ystad, S. & Kronland-Martinet, R. Eluding the Physical Constraints in a Nonlinear Interaction Sound Synthesis Model for Gesture Guidance. *Applied Sciences* **6**, 192 (2016).
